# On protein abundance distributions in complex mixtures

**DOI:** 10.1186/1477-5956-11-5

**Published:** 2013-01-29

**Authors:** JA Koziol, NM Griffin, F Long, Y Li, M Latterich, JE Schnitzer

**Affiliations:** 1 The Scripps Research Institute, 10550 N Torrey Pines Road, La Jolla, CA, 92037, USA; 2 Proteogenomics Research Institute for Systems Medicine, 11107 Roselle Street, San Diego, CA, 92121, USA

## Abstract

Mass spectrometry, an analytical technique that measures the mass-to-charge ratio of ionized atoms or molecules, dates back more than 100 years, and has both qualitative and quantitative uses for determining chemical and structural information. Quantitative proteomic mass spectrometry on biological samples focuses on identifying the proteins present in the samples, and establishing the relative abundances of those proteins. Such protein inventories create the opportunity to discover novel biomarkers and disease targets. We have previously introduced a normalized, label-free method for quantification of protein abundances under a shotgun proteomics platform (Griffin et al., 2010). The introduction of this method for quantifying and comparing protein levels leads naturally to the issue of modeling protein abundances in individual samples. We here report that protein abundance levels from two recent proteomics experiments conducted by the authors can be adequately represented by Sichel distributions. Mathematically, Sichel distributions are mixtures of Poisson distributions with a rather complex mixing distribution, and have been previously and successfully applied to linguistics and species abundance data. The Sichel model can provide a direct measure of the heterogeneity of protein abundances, and can reveal protein abundance differences that simpler models fail to show.

## Introduction

Large-scale proteome analysis using mass spectrometry and subcellular fractionation techniques can provide inventories of proteins identified in organelles, cells and tissues (e.g., [1-3]). Such protein inventories create the opportunity to discover novel biomarkers and disease targets (e.g., [4-7]). But a more detailed description of cells, tissues and organisms in health and disease would benefit greatly from quantitative tools that can carefully and comprehensively quantify the individual building blocks, which comprise the living entity. The ability to quantify properly identified proteins in biological samples in a comprehensive fashion engenders an enhanced understanding of cellular behavior during development or in response to disease, and can lead to novel biomarker and target discoveries [4,8].

Much effort has gone into developing more accurate and cost effective technologies that can capture the dynamics of biomolecular diversity in more quantitative ways. While significant advances have been made to develop accurate genomic sequencing tools [9] and highly accurate gene expression analytical methods [10], reliable methods of quantifying protein expression and modification levels have been challenging [11].

This difficulty is in part due to the immense chemical complexity of proteins, which are made up from over twenty amino acid monomers with distinct chemical properties, as contrasted to biopolymers such as RNA that are constituted from four monomers with similar properties. Currently there are no feasible direct methods to establish protein sequences like that of nucleotide polymers; the only method to directly determine the identity and the quantity of proteins in a mixture in large scale is the mass spectrometer, which can determine peptide sequences based on fragmentation pattern analysis and expression levels via direct or indirect means of analysis.

Quantitative proteomic mass spectrometry is indispensable to providing valuable insights into protein content and activity in various cellular states. There are at present three principal methods of quantifying proteins via mass spectrometry: labeling approaches such as iTRAQ and SILAC, which aim to reduce experimental variance and allow relative comparison of peptides between samples [12,13]; absolute quantitative approaches such as MRM and SISCAPA [7,14], which are highly accurate but thus far at the expense of completeness; and, label free approaches that rely on counting spectra or peptide numbers as a proxy for expression level (reviewed in [15]), or on ion intensities [16], or that jointly consider peptide count, spectral count, and fragment-ion intensity [17]. The latter method is particularly well suited for comparing clinical specimens for biomarker identification where samples are collected over long time periods and may have to be compared across sites [6,18].

We have previously introduced a normalized, label-free method for quantification of protein abundances under a shotgun proteomics platform [17]. The introduction of this method for quantifying and comparing protein expression leads naturally to the issue of modeling protein abundances. In this note, we examine various models for patterns of relative protein abundance from typical 2 dimensional liquid chromatography mass spectrometry (2D-LC-MS/MS) experiments.

Characterization of the joint distribution of all protein abundances in a proteome is complicated by the fact that protein abundances typically differ over several orders of magnitude. As might be expected, this joint distribution can be rather complex, and we would not expect a Gaussian distribution would adequately characterize it [17,19]. Here, we make no Gaussian assumptions about any abundances. Rather, from a somewhat historical perspective, we have chosen distributions that have been proposed for modeling word counts and species abundances, as we are positing an analogous problem to these precedents. We formally compare different families of distributions for protein abundance, with goodness of fit criteria utilized to determine adequacy of the models for summarizing the underlying data. Our fitting criteria allow us to determine which models best capture the underlying data structure, and would be appropriate for characterizing protein abundance distributions.

The protein abundance distributions can be utilized to establish the success rate of the experiments as defined by Eriksson and Fenyo [19], or what we have referred to as coverage [20]. Our ultimate goal was to identify a distribution that would improve the quantitative accuracy of label-free stochastic mass spectrometry.

## Methods

### Sample preparation

Luminal vascular endothelial cell plasma membranes and their caveloae were directly isolated from rat lung as previously described [21,22]. Proteins were pre-fractionated on SDS-PAGE gels prior to 2 dimensional liquid chromatography mass spectrometry (2D-LC-MS/MS). Gel lanes were cut into slices, approximately 50 per lane, for in-gel proteolytic digestions. Digested peptides were extracted from each gel slice three times with 20% ACN and 10% formic acid solution. The peptides extracted from each gel slice were first pooled into 7 groups then lyophilized. Each sample, either plasma membrane (experiment 1) or caveolae (experiment 2) was separated into five different gel lanes, and each lane was subjected to a complete 2D-LC-MS/MS analyses resulting in five replicate MS analyses of each sample. Proteins were inferred from each replicate [with the implication, that some proteins were not observed in every replicate]. By convention, we dropped from consideration any proteins detected in one run only.

### Mass spectrometry

*2DLCMS*/*MS:* Lyophilized peptides were resuspended with 15 μl of buffer A (0.1% formic acid, 5% Acetonitrile (ACN)), then loaded onto a two-dimensional microcapillary column (manually packed C_18_ reversed phase and strong cation exchange column). The loaded samples were directly introduced into the LTQ mass spectrometer equipped with ESI nanospray ion source by eluting the bound peptides with a 2D-LC-MS/MS scheme controlled by Agilent 1100 HPLC quaternary pump [3]. Briefly, 17 salt steps (ammonium acetate) were applied. Each salt step was followed by a 5 to 80% ACN gradient containing 0.1% formic acid to elute the peptides on the C_18_ column. The flow rate was maintained at 200 to 250 nl/min.

Data acquisition for the LTQ was carried out in data-dependent mode. Full MS scans were recorded on the eluting peptides over the 400–1400 m/z range with one MS scan followed by three MS/MS scans of the most abundant ions. The temperature of the ion transfer tube of both mass spectrometers was set at 180°C and the spray voltage was 2.0 kv. The normalized collision energy was set at 35%. A dynamic exclusion was applied for Repeat Count of 2, a Repeat Duration of 0.5 minute, and an Exclusion Duration of 10 min.

### Database search for protein identification

The acquired MS/MS spectra were converted into mass lists using the Extract_msn program from Xcalibur and searched against a protein database containing rat sequences using the Sequest program in the Bioworks™ 3.1 for Linux (Thermo Fisher Scientific, Inc., Waltham, MA, USA). The searches were performed allowing for tryptic peptides only with peptide mass tolerance of 1.5 Da and a minimum of 21 fragmented ions in one MS/MS scan. Accepted peptide identification was based on a minimum Cn score of 0.1; minimum cross correlation score of 1.8(z=1), 2.5(z=2), 3.5(z=3). False positive identification rate was determined by the ratio of number of peptides found only in the reversed database to the total number of peptides found in both forward and reverse databases. The false positive identification rates were ≤ 1%. The positive protein identification results were extracted from Sequest.out files, filtered and grouped with DTASelect software using above criteria. Proteins were identified based on 2 unique significantly identified peptides.

### Statistical methods

We consider the following discrete probability distributions:

(1) The negative binomial (NB) distribution, with probability mass function

$$\;\begin{array}{c} P_{\mathrm{nb}}\left(k;\gamma ,p\right)=\frac{\Gamma \left(\gamma +k\right)}{k!\Gamma \left(\gamma \right)}p^{k}{\left(1-p\right)}^{\gamma },k\\ \;=0,1,\ldots ,\gamma >0,0<p<1. \end{array}$$

(2) The discrete Weibull distribution, with probability mass function

$$\;\begin{array}{c} \mathrm{Pw}\left(k;v,p\right)=p^{k^{v}}-p^{{\left(k+1\right)}^{v}},k=0,1,\ldots ,v>0,0\\ \;<p<1. \end{array}$$

(3) The Zipf distribution, with probability mass function

$$\;P_{z}\left(k;p\right)=\frac{k^{-\left(1+\rho \right)}}{\mathrm{Zeta}\left(1+\rho \right)},k=1,2,\ldots ,\rho >0\text{,}$$

where Zeta(.) is Riemann’s zeta function.

(4) The Zipf-Mandelbrot distribution, with probability mass function

$$\;\begin{array}{c} P_{\mathrm{zm}}\left(k;\rho ,a\right)=\frac{{\left(k+a\right)}^{-\left(1+\rho \right)}}{\mathrm{Zeta}\left(1+\rho ,a\right)},k=1,2,\ldots ,\rho \\ \;>0,a>0. \end{array}$$

where here Zeta(r,a) denotes the Hurwitz zeta function.

(5) The Sichel distribution, with probability mass function

$$\begin{array}{c} P_{s}\left(k;\alpha ,\theta ,\gamma \right)=\frac{{\left(1-\theta \right)}^{\gamma /2}}{K_{\gamma }\left(\alpha \sqrt{1-\theta }\right)}\frac{{\left(\alpha \theta /2\right)}^{k}}{k!}K_{k+\gamma }\left(\alpha \right),\\ \;k=0,1,\ldots ,\alpha >0,0<\theta <1,-\infty <\gamma <\infty \end{array}$$

where K_γ_(z) denotes the modified Bessel function of the second kind of order γ and argument z.

(6) The Poisson inverse Gaussian (PIG) distribution. This is a special case of the Sichel distribution, obtained by setting γ = −1/2 in the probability mass function P_s_. [Numerical evaluation of K_γ_(z) is enormously simplified if γ = −1/2 or differs from −1/2 by an integer, advantageous in an earlier era of less powerful computational capabilities].

Our choice of these distributions is based partly on historical considerations, as we now describe.

The Poisson distribution is a standard baseline model for discrete data, and is often used as a starting point for deriving more realistic models that meet the characteristics of an observed set of data. Mathematically, the Poisson is a one-parameter distribution, with the mean equal to the variance. If discrete data show overdispersion relative to the Poisson, generalizations might be introduced to accommodate this. Greenwood and Yule [23] suggested a model in which the mean in the Poisson distribution is itself random, following a gamma distribution. This leads to a two-parameter distribution, the negative binomial, for discrete data. In turn, the negative binomial has become a standard baseline model for discrete data overdispersed relative to the Poisson.

In a seminal article, Fisher and colleagues [24] introduced the notion of mathematically modeling species abundance data. Their motivation was to model butterfly abundance data from Malaya [25], and Fisher explored the truncated negative binomial distribution and extensions to this end. With species abundance data, as with our peptide setting, one must consider the zero-truncated forms of the underlying distributions, to accommodate the fact that certain species may not be observed in a finite sampling frame. This can lead to some added complexities relative to model fitting, as for example, described by Sampford [26] relative to the truncated negative binomial distribution. As with Greenwood and Yule, Fisher et al. [24] assumed that abundances could be modeled by a gamma distribution, which led to the negative binomial. A special case is Fisher’s log-series model, where the shape parameter of the gamma distribution tends to zero. Engen [27] provides a comprehensive review of species abundance models in ecology.

The eponymous Zipf’s law was introduced by Zipf [28] as a word frequency distribution: if one tabulates from an arbitrary text the number of words arranged in the order of their frequency of usage, the resulting word frequency distribution is generally reverse J-shaped, with a very long upper tail. Zipf’s law is a mathematical power-law representation of this type of distribution. Zipf’s frequency distribution was later generalized by Mandelbrot [29], again in a linguistics context.

The discrete Weibull [30] is another model for skewed, power-like discrete data. The incorporation of an additional parameter, as with Zipf-Mandelbrot, allows added flexibility, to accommodate situation in which the power-law relationship tends to decay in the tail. This is closely related to the stretched exponential distribution [31]. Newman [32] and Clauset et al. [33] give particularly lucid accounts of power-law distributions.

The Sichel distribution was introduced by Holla [34], and popularized in a series of papers by Sichel (e.g., [35-38]). Sichel and others have applied it both to linguistics and to species abundance data (e.g., [39]). The special case of an inverse Gaussian mixing distribution, leading to the Poisson inverse Gaussian distribution, enjoys some computational advantages (e.g., [40]). The Sichel distribution is a mixed Poisson distribution, and can be generalized by using mixing distributions other than the inverse Gaussian (e.g., [41-44]).

From a theoretical perspective, the negative binomial and Sichel distributions are attractive models for protein abundance data. The frequencies of the different proteins in the sample can be taken as independent Poisson variables, where the Poisson parameters are heterogeneous; a mixing distribution should then be chosen to accommodate the overdispersion. In this regard, the Poisson inverse Gaussian distribution seems preferable to the negative binomial, but the Sichel distribution, with one additional free parameter relative to the Poisson inverse Gaussian distribution, is correspondingly even more flexible.

We used maximum likelihood techniques for fitting observed protein abundance data to all models: this typically provides more efficient and robust estimates than other methods, developed prior to the advent of inexpensive computing resources. Goldstein et al. [45] have cautioned against informal methods of parameter estimation with power-law based discrete distributions, and Clauset et al. [33] provide theoretical justification for maximum likelihood. We utilized Mathematica 8.0 (Wolfram Research, Inc., 2010) for numerical fitting using its default global optimization algorithm; in addition, the program also provides built-in numerical evaluation of the special functions incorporated in the probability mass functions above, which facilitates the optimization.

The method of maximum likelihood in our setting is straightforward. We describe the method generically, as follows. Let X denote a positive-integer valued random variable, with Prob(X=i)=P(i;θ) for some vector of parameters θ. We draw a finite random sample, and observe X=i with frequency f_i,_ for i=1,2,…,m. The method of maximum likelihood entails finding the vector $\hat{\theta }$ that maximizes the log of the likelihood function

$$\;\mathrm{LL}=\sum_{i=1}^{m}f_{i}\log\left[P\left(i;\theta \right)\right]\text{.}$$

[In practice it is generally more convenient to maximize the log of the likelihood function than the likelihood itself]. With our data, the X_i_ are the various protein abundances, and the P(i) are the probabilities determined from the models given above. Note, however, that the minimal observed protein abundance is 1, whereas the supports of the negative binomial, discrete Weibull, and Sichel distributions begin at 0. Hence for these distributions, we fit zero-truncated forms of the distributions: when maximizing the log likelihood for these distributions, the P(i) are replaced by P(i)/(1-P(0)) in the above formula for *LL*. The supports for the Zipf and Zipf-Mandelbrot distributions begin at 1, obviating the need to deal with truncated forms of these distributions.

Because the models are not always nested, we adopt the Akaike information criterion (AIC; [46]) as our general criterion for comparing models. [In the case of nested models, as with the Zipf nested within the Zipf-Mandelbrot, one might use a likelihood ratio test, to assess the relative improvement in fit with the more complex model relative to the simpler one.] The AIC value is defined as −2[log likelihood - # fitted parameters]. Given a set of potential models for the data, the minimum AIC value would be indicative of the preferred model. We remark that, there is one fitted parameter for the Zipf distribution, two fitted parameters for the negative binomial, discrete Weibull, Zipf-Mandelbrot, and Poisson inverse Gaussian distributions, and three fitted parameters for the Sichel distribution.

We display observed and fitted distributions with rank-frequency plots [47]. The rank-frequency plot of a frequency distribution is in log-log coordinates, with x denoting the ranks of the items in the distribution, and y the corresponding relative frequencies. [A Zipf distribution would be a straight line in a rank-frequency plot, and the plot can be utilized to estimate the parameter r characterizing the Zipf distribution]. Newman [32] describes these plots in greater detail, and astutely notes their equivalence to complementary cumulative distribution function plots, but with log-log and not linear coordinates. We utilize Newman’s construction in the following. Specifically, we start with a listing of all the proteins, along with their frequency of occurrence (abundance), ranked in order of increasing abundance. The complementary cumulative distribution P(x) of the frequency x is defined as the fraction of proteins with abundance greater than or equal to x. Our plots depict both the observed and the fitted complementary cumulative distributions.

## Results

We are interested in quantitatively mapping the proteins expressed on the surface of vascular endothelial cells as they exist natively in tissue, and have developed subcellular tissue fractionation techniques to isolate luminal endothelial cell surface membranes directly from lung and other tissues. These endothelial plasma membranes (experiment 1) and their caveolae (experiment 2) were isolated from rat organs, and were subsequently analyzed by SDS-PAGE and mass spectrometry (see Methods). With the first experiment, a total of 27252 peptides were detected in 5 2D-LC-MS/MS cycles; these identified 2075 unique proteins, based on our model selection criteria outlined in the methods section. In the second experiment, a total of 13226 peptides were detected in 5 2D-LC-MS/MS cycles; these identified 1069 unique proteins. Summary statistics for the relative peptide counts are given in Table 1. If abundances were Poisson distributed, then the ratio of variance to mean would be about 1; the large variance/mean ratios are indicative of extra-Poisson variability. Within each experiment, the data are quite dispersed, and extremely right-skewed; heavy tails exist because of several extreme values of abundance counts.

**Table 1:** Summary statistics for peptide counts

	**Min**	**Max**	**Median**	**Mean**	**SD**	**Skewness**	**Kurtosis**	**Var/Mean**
**Expt 1**	1	525	7	13.13	26.36	10.56	164.7	52.9
**Expt 2**	1	302	6	12.37	20.43	6.06	60.8	33.7

Caption. Experiments 1 and 2 refer to the membrane replicates and caveolae replicates respectively, as described in the methods. 2075 unique proteins were identified in experiment 1 and 1069 in experiment 2.

**Figure 1:**
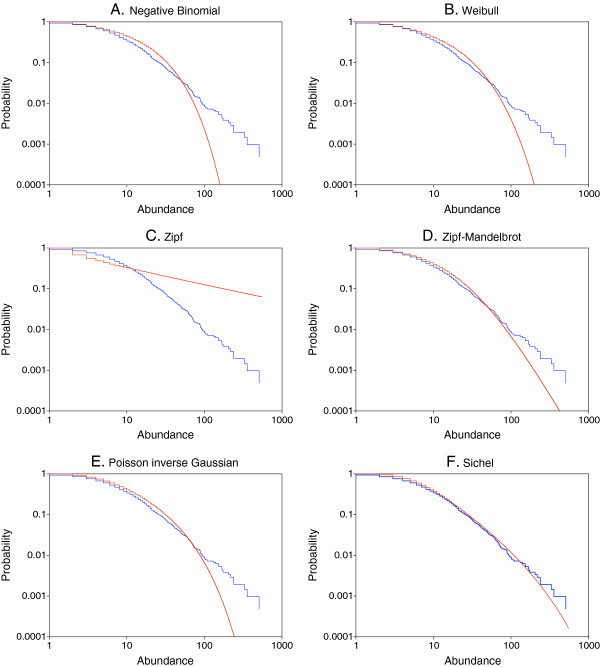
**Rank-frequency plots of protein abundances from the first experiment, together with fitted distributions.A**. Negative binomial. **B**. Discrete Weibull. **C**. Zipf. **D**. Zipf-Mandelbrot. **E**. Poisson inverse Gaussian. **F**. Sichel. Observed data are depicted in blue, and the fitted distributions are depicted in red. As described in the Methods, we start with a listing of all the proteins, along with their frequency of occurrence (abundance). The complementary cumulative distribution P(x) of the abundance x is defined as the fraction of proteins with abundance greater than or equal to x. Our plots depict both the observed and the fitted complementary cumulative distributions (ordinates) vs protein abundances (abscissas).

**Figure 2:**
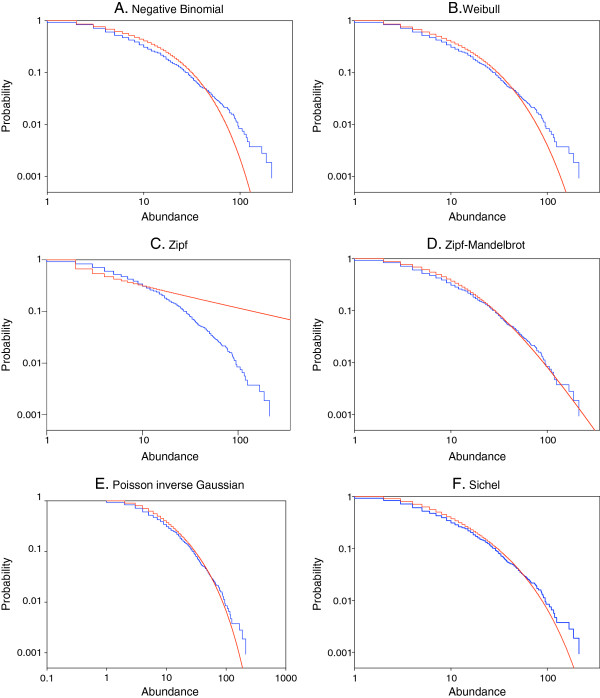
**Rank-frequency plots of protein abundances from the second experiment, together with fitted distributions.A**. Negative binomial. **B**. Discrete Weibull. **C**. Zipf. **D**. Zipf-Mandelbrot. **E**. Poisson inverse Gaussian. **F**. Sichel. Observed data are depicted in blue, and the fitted distributions are depicted in red. As described in the Methods, we start with a listing of all the proteins, along with their frequency of occurrence (abundance). The complementary cumulative distribution P(x) of the abundance x is defined as the fraction of proteins with frequency greater than or equal to x. Our plots depict both the observed and the fitted complementary cumulative distributions (ordinates) vs protein abundances (abscissas).

In Figures 1 and 2 we display rank-frequency plots of the observed protein abundance distributions from the two experiments, along with the individual models fitted by maximum likelihood. The AIC values corresponding to the fits are given in Table 2. With both experiments, the ordering of the models would be

$$\begin{array}{c} \mathrm{Sichel}<\mathrm{PIG}<\mathrm{discrete}\;\mathrm{Weibull}<\mathrm{NB}\\ \;<\mathrm{Zipf}-\mathrm{Mandelbrot}<\mathrm{Zipf}\text{,} \end{array}$$

with the left to right ordering indicative of best to worst fitting. The added flexibility of the general Sichel distribution with arbitrary parameter γ provides an improvement in fit over the Poisson-inverse Gaussian distribution with γ = −1/2; in turn, both of these models are noticeably better than the other models, relative to AIC values. The rank-frequency plots for Set 1 show that only the Sichel model adequately represents the empirical frequency distribution in the right tail. With Set 2, the Poisson-inverse Gaussian model more closely resembles the Sichel model; lack of fit in the right tail is again noticeable for the other models. The Zipf distribution is particularly noteworthy for its lack of fit throughout the range of the empirical distribution of frequencies.

**Table 2:** Comparative statistics for six models

**Model**	**AIC, Expt 1**	**AIC, Expt 2**
**negative binomial**	14533.9	7326.9
**discrete Weibull**	14413.6	7280.8
**Zipf**	16146.9	8067.0
**Zipf-Mandelbrot**	14703.5	7482.7
**Poisson inverse Gaussian**	14238.4	7203.0
**Sichel**	14167.3	7189.8

Caption. Experiments 1 and 2 refer to the membrane replicates and caveolae replicates respectively, as described in the methods. AIC denotes Akaike’s information criterion; smaller values connote better model fits.

## Discussion

It has become apparent that peptide and thus protein abundances, as measured by large scale high-throughput shotgun proteomics experiments, are not normally distributed [17,19]. This may be reflective of the complex nature of the proteome, especially when post-translational modifications are taken into account, or the inherent sampling limitations of the currently available MS technology as mentioned in the introduction. Nonetheless, we sought to characterize the protein abundance distributions in terms of their contributing peptides from two separate large-scale 2D-LC-MS/MS protein identification experiments. Our goal was to identify a distribution model that best fits or describes the protein abundance data, which can take into account the real world variation in protein abundances.

From the earliest reports of 2D-LC-MS/MS data [14,48,49], it has become clear that protein abundance differs over several orders of magnitude, with many proteins having a relatively small abundance, a few with relatively large abundances. This reflects the inherent dynamic range of any proteome, prior to identification by mass spectrometry. One must not forget that protein detection by traditional mass spectrometry methods is dependent on the inherent physical properties of the proteins and their resulting peptides. Peptide detection is highly dependent on the ease with which the peptide can be ionized. Ionization efficiency can be thought of as the tendency of the peptide to ionize and contribute to a mass spectrum thus facilitating the identification of the peptide and thus the protein. This is influenced mainly by the inherent structural properties of the peptide, such as length, mass, amino acid composition, and various biophysical properties, such as hydrophobicity, number of charges and potential modifications. Thus, one must be acutely aware that not every peptide in a given complex sample can and will be identified even though multiple methods have been developed in recent years to enhance peptide and protein coverage of a complex protein sample [3,50].

Let us next consider the issue of the external validity (generalizability) of our findings. To address this, we analyzed a smaller dataset reported by Ishihama et al. [51], Table 1. The relevant data consist of concentrations of 46 proteins that the authors had identified and quantified in mouse neuro2a cells [with a different quantitation method than that of Griffin et al.]. We proceeded to fit the 6 distributions described previously, and obtained the following ordering of the models:

Sichel < PIG < Zipf-Mandelbrot < discrete Weibull < NB < Zipf.

The respective AIC values were: 586.97, 592.45, 599.53, 603.54, 604.59, and 705.35. The pre-eminence of the Sichel distribution remains, as does the poor performance of the Zipf distribution. With this smaller dataset, Zipf-Mandelbrot outperforms the discrete Weibull and the negative binomial, although differences are at best modest. Nevertheless, we have insufficient evidence that a Sichel distribution would obtain with other quantification methods (e.g., spectral counting methods emPAI or RIBAR / xRIBAR); a cautious interpretation is, that we observed a Sichel distribution with the quantification method of Griffin et al. [17], but that the observed distribution may also depend on the mass spectrometer technology used.

From the analyses described in this study, one might infer that simple models of protein distribution do not adequately fit the experimental data, with empirical evidence pointing toward a more complicated mixing distribution. Indeed, the more complex Poisson inverse Gaussian or Sichel distributions work well to accommodate the heavy tail that is typically observed in proteomics experiments. These models accommodate the fact that protein abundances as reflected in the number of peptides detected per protein within a given sample and between identical samples can be different. This is not surprising giving the complex nature of the sample and the contribution of ion suppression effects which can mean that a peptide detected in one sample may not be detected in a subsequent MS analysis of the same sample. In fact, we previously found that each MS measurement of a shotgun proteomics analysis identifies only a subset of proteins and that second and third MS measurements of the same sample would reveal about 33% and 16% respectively of new proteins not detected in the previous analyses [1,20]. This means that multiple MS measurements should be performed to comprehensively define the full proteome to the degree possible with the technique used, hence why 5 replicate analysis of each sample were performed in the protein identification experiments analyzed in this paper. Furthermore, due to the intrinsic properties of some proteins, especially their large hydrophobicity peptides, or lack of accessible tryptic cleavage sites, some peptides may never be detected by the mass spectrometer. This suggests that, rather than total proteomic identification, the goal of these experiments should be adequate coverage of the entire proteome [20]. Thus, the ability to model protein abundance distributions from 2D-LC-MS/MS experiments or even fit the distributions to a specific model implies that one could theoretically exploit the properties of the model to improve protein coverage through optimizing experimental design [20].

## Competing interests

The authors declare no competing financial interests.

## Authors’ contributions

The study was conceived by JAK, NMG, ML, and JES. NMG, FL, YL performed the sample preparation and mass spectrometry experiments. JAK and NMG undertook subsequent analyses. JAK, NMG, ML, and JES prepared the manuscript. All authors contributed to manuscript editing. All authors read and approved the final manuscript.
